# Current and Emerging Therapies in Primary Vitreoretinal Lymphoma: A Narrative Review

**DOI:** 10.3390/cancers18172865

**Published:** 2026-09-04

**Authors:** Mihai-Luca Cioboată, Suher Abduraman, Ioana Tofolean, Radu Burcea, Miruna Cioboată, Ali Rıza Cenk Çelebi, Florian Baltă

**Affiliations:** 1 Department of Ophthalmology, Carol Davila University of Medicine & Pharmacy, 020021 Bucharest, Romania; mihai-luca.cioboata@drd.umfcd.ro (M.-L.C.); yoana_teo@yahoo.com (I.T.); florian.balta@umfcd.ro (F.B.); 2 Department of Ophthalmology, Clinical Institute of Ophthalmological Emergencies “Prof. Dr. Mircea Olteanu”, 010464 Bucharest, Romania; abduramansuher@yahoo.com; 3 Department of Ophthalmology, Istinye University School of Medicine, 34396 Istanbul, Türkiye; arcenkcelebi@gmail.com

**Keywords:** primary vitreoretinal lymphoma, intravitreal methotrexate, MYD88 mutation, interleukin-10, Bruton’s tyrosine kinase inhibitors

## Abstract

This review aims to provide a comprehensive overview of the currently available therapies for primary vitreoretinal lymphoma (PVRL) and to evaluate emerging therapeutic strategies that may improve patient outcomes. A comprehensive literature search was conducted in databases including PubMed/MEDLINE, Embase, and Scopus for studies published up to 2026. Search terms included “primary vitreoretinal lymphoma”, “intravitreal methotrexate”, “rituximab”, “radiotherapy”, “targeted therapy”, and “immunotherapy”. Eligible publications included randomized controlled trials, prospective and retrospective studies, observational studies, systematic reviews, relevant clinical guidelines, case series, and case reports. Studies were independently screened and assessed by two reviewers. Current therapeutic strategies include intravitreal chemotherapy, systemic high-dose methotrexate-based regimens, and radiotherapy, either as monotherapy or in combination. While intravitreal approaches provide effective local disease control, they fail to address occult or subsequent central nervous system (CNS) dissemination, necessitating the use of systemic treatments in selected patients. In recent years, significant progress in the understanding of PVRL pathophysiology, including the role of MYD88 mutations and interleukin-10 signaling, has paved the way for the development of targeted and immunomodulatory therapies. Agents such as Bruton’s tyrosine kinase inhibitors, immunomodulatory drugs, and immune checkpoint inhibitors have demonstrated promising results in early clinical studies, particularly in relapsed or refractory disease. Despite advances in diagnostic techniques, the management of PVRL remains challenging due to its heterogeneous presentation, high relapse rates, and frequent progression to CNS involvement.

## Simple Summary

Primary vitreoretinal lymphoma (PVRL) is a rare and aggressive eye cancer that can also affect the brain. The review discusses current treatments, including intravitreal methotrexate and rituximab, systemic chemotherapy, and radiotherapy. Treatment choice depends on whether the disease is limited to the eye or involves the central nervous system. New targeted and immune-based therapies are also being studied. Overall, PVRL remains challenging to manage, and further research and clinical trials are needed to establish safer, more effective, and individualized treatment strategies.

## 1. Introduction

Primary vitreoretinal lymphoma (PVRL) is a rare, high-grade extranodal lymphoma that predominantly arises from diffuse large B-cell lymphoma (DLBCL) and is considered part of the spectrum of primary central nervous system lymphoma (PCNSL). The disease primarily involves the vitreous, retina, and subretinal space, often masquerading as chronic uveitis, which contributes to delayed diagnosis and management [1].

The diagnosis of PVRL remains challenging and typically requires a combination of clinical suspicion, cytological analysis, immunohistochemistry, flow cytometry, and molecular testing [2]. Elevated intraocular interleukin-10 (IL-10) levels and an IL-10/IL-6 ratio greater than 1 are considered supportive of PVRL, although these biomarkers should not be interpreted independently of cytopathological and clinical findings. The presence of MYD88 mutations, particularly MYD88 L265P, has emerged as a valuable diagnostic marker and provides further insight into disease pathogenesis [3]. This mutation can be detected by allele-specific PCR or droplet digital PCR in vitreous samples and, increasingly, in cell-free DNA obtained from the aqueous humor. Targeted next-generation sequencing enables broader molecular profiling, including the identification of recurrent alterations in MYD88, CD79B, PIM1, and other genes involved in B-cell receptor and NF-κB signaling. Liquid-biopsy approaches using aqueous or vitreous humor may therefore complement conventional diagnostic methods and facilitate repeated, minimally invasive molecular assessment. These molecular features may also inform the selection of targeted therapies, particularly inhibitors of Bruton tyrosine kinase, underscoring the shift toward more personalized treatment approaches [1,3]. Furthermore, preliminary evidence suggests that serial measurement of IL-10 and monitoring of tumor-derived cell-free DNA may contribute to the assessment of treatment response and the early detection of recurrence, although these applications remain investigational and require prospective validation.

Despite advances in diagnostic modalities, the optimal management of PVRL remains controversial [4]. Current treatment strategies include local therapies, such as intravitreal methotrexate and rituximab, as well as systemic chemotherapy and radiotherapy [5]. However, no standardized treatment guidelines exist, and therapeutic decisions are often based on institutional protocols and individual patient characteristics [5,6]. Moreover, high rates of relapse and treatment-related toxicity continue to pose significant clinical challenges [7]. Recent advances in the understanding of the molecular and immunological pathways involved in PVRL pathogenesis have stimulated the development of innovative therapeutic strategies, including targeted agents and immune-based approaches [8,9]. These novel modalities have shown considerable potential for enhancing disease control while minimizing treatment-related toxicity, particularly in patients with relapsed or refractory disease [10].

The aim of this review is to provide a comprehensive overview of current and emerging therapies in PVRL, critically evaluating their clinical utility, limitations, and future potential. Accordingly, this review focuses on recent therapeutic advances and examines their potential within personalized management strategies, while underscoring the need for evidence-based and standardized treatment recommendations.

## 2. Materials and Methods

A comprehensive literature search was conducted in databases including PubMed/MEDLINE, Embase, and Scopus for studies published up to 2026. The search combined terms related to vitreoretinal lymphoma (“primary vitreoretinal lymphoma”, “vitreoretinal lymphoma”, “ PVRL”, and “PIOL”) with treatment-related term, including “intravitreal methotrexate”, “methotrexate”, “rituximab”, “radiotherapy”, “systemic chmeotherapy”, “targeted therapy”, “stem cell transplantation”, “BTK inhibitor”, “lenalidomide”, “CAR-T”, and “immunotherapy”. Studies were eligible if they included adult patients with isolated PVRL or PVRL associated with primary central nervous system lymphoma (PCNSL), evaluated a local, systemic, combined, maintenance, or emerging therapy, and reported relevant therapeutic outcomes. Eligible publications included randomized controlled trials, prospective and retrospective studies, observational studies, systematic reviews, relevant clinical guidelines, case series, and case reports. Because of the rarity of PVRL, case reports were included when they described novel treatments for which larger studies were unavailable. Studies involving PCNSL without documented ocular involvement were considered only when they evaluated emerging therapies with potential relevance to PVRL and were analyzed separately from PVRL-specific evidence. Studies focused exclusively on uveal, conjunctival, orbital, or other non-vitreoretinal lymphomas, preclinical studies, editorials, and publications without sufficient therapeutic data were excluded.

Studies were independently screened and assessed by two reviewers, and relevant data on therapeutic efficacy, safety, and emerging treatment strategies were qualitatively synthesized. Any discrepancies in study selection were resolved through consensus discussions, with involvement of a third reviewer when necessary. The database searches retrieved 855 records, and an additional 12 publications were identified by manually screening the reference lists of relevant articles. Following the removal of 307 duplicates, 560 unique records were screened by title and abstract, of which 444 were excluded. The full texts of the remaining 116 articles were assessed for eligibility. Seventy-five articles were subsequently excluded because they focused primarily on diagnostic aspects, lacked PVRL-specific therapeutic outcomes, or included patients with PCNSL without separately reported ocular data. Consequently, 41 studies fulfilled the eligibility criteria and were included in the final scoping review. Because of substantial heterogeneity in study populations, treatment protocols, response definitions, and follow-up duration, a quantitative meta-analysis was not performed. Evidence was synthesized narratively according to treatment category: intravitreal therapy, ocular radiotherapy, systemic chemotherapy, combined treatment, autologous stem cell transplantation, targeted therapies, and emerging immunotherapies. For each therapeutic modality, ocular efficacy, CNS outcomes, toxicity, level of evidence, and principal limitations were evaluated. ChatGPT (GPT-5; OpenAI, San Francisco, CA, USA) was utilized exclusively for language refinement and editing purposes. All scientific content, data interpretation, and conclusions were developed independently by the authors.

## 3. Current Treatment Modalities

The optimal management of PVRL remains controversial because the disease-specific evidence is of low certainty. Most published data derive from retrospective, uncontrolled, single-center or multicenter cohorts and small case series [11,12]. Although prospective single-arm and early-phase studies have evaluated selected intravitreal, systemic, and targeted therapies, they have generally enrolled small numbers of patients and frequently included heterogeneous populations, combining newly diagnosed and relapsed or refractory disease, unilateral and bilateral ocular involvement, and isolated ocular disease with concomitant CNS lymphoma. To date, no completed randomized controlled trial has established the superiority of local, systemic, or combined treatment as the first-line strategy specifically for PVRL.

The available studies are subject to several important sources of bias. Selection bias and confounding by indication are particularly relevant because younger and medically fit patients, as well as those with bilateral ocular disease or documented CNS involvement, are more likely to receive systemic or combined therapy. Referral bias, incomplete retrospective data collection, non-masked assessment of ocular response, variable follow-up, and publication bias may further influence the reported outcomes. Moreover, the small patient cohorts generate imprecise effect estimates and limit adequately adjusted subgroup analyses. Considerable heterogeneity exists regarding intravitreal injection schedules, systemic chemotherapy regimens, radiotherapy doses, consolidation strategies, response definitions, and surveillance protocols. These limitations restrict direct comparisons between studies and prevent firm conclusions regarding the effect of treatment on CNS dissemination, progression-free survival, or overall survival. In particular, high rates of initial ocular remission should not be interpreted as evidence of CNS protection or improved survival.

The updated EANO recommendations and the 2024 EHA–ESMO Clinical Practice Guideline recognize PVRL as part of the PCNSL spectrum while acknowledging the limited PVRL-specific evidence. The EHA–ESMO guideline recommends HD-MTX-based induction chemotherapy for medically fit patients with PVRL, using regimens employed for PCNSL; however, this recommendation is supported by level IV evidence and carries a grade C strength of recommendation. Intravitreal MTX may be added when rapid control of intraocular disease is required, likewise on the basis of level IV, grade C evidence. Local therapies, including intravitreal MTX and ocular radiotherapy, remain acceptable first-line options for patients who are unfit for systemic chemotherapy or have relevant contraindications, supported by level IV, grade B evidence [12,13]. In selected patients with isolated unilateral ocular disease, local treatment alone may also be considered after multidisciplinary discussion, although it does not address occult CNS disease and requires close neurological surveillance. Conversely, bilateral ocular involvement generally favors consideration of systemic therapy, with additional local treatment when rapid, sight-preserving ocular control is necessary. When CNS or cerebrospinal fluid involvement is documented, treatment should follow a PCNSL-directed HD-MTX-based systemic strategy, with intravitreal therapy added for persistent, extensive, or sight-threatening ocular disease.

Accordingly, treatment selection should be individualized within a multidisciplinary team involving ophthalmology, hematology, neuro-oncology, and radiation oncology. Relevant factors include the presence or absence of CNS disease, unilateral versus bilateral ocular involvement, age, frailty, performance status, renal function, comorbidities, previous treatment, anticipated ocular and systemic toxicities, patient preferences, and institutional expertise.

### 3.1. Intravitreal Therapy

#### 3.1.1. Methotrexate

Methotrexate (MTX) (400 µg/0.1 mL) remains the most extensively studied agent and is commonly administered according to “Intensive-Consolidation-Maintenance” protocols (Table 1) [13,14]. In a landmark series by Frenkel et al. [15], intravitreal MTX achieved complete ocular remission in the majority of treated eyes, with a low rate of intraocular recurrence, establishing it as an effective first-line local therapy. In their protocol, MTX was administered intravitreally twice weekly for 4 weeks, followed by once-weekly injections for 8 weeks and subsequently once monthly for 9 months, resulting in a total of 25 injections [15]. A more recent modification of the intravitreal MTX regimen was proposed by Zhou et al. [16], aiming to reduce the treatment burden while maintaining therapeutic efficacy. Compared with the classical protocol described by Frenkel et al. [15], which requires 25 injections over one year, the modified “Intensive-Consolidation-Maintenance” schedule consists of weekly injections for one month, followed by biweekly injections for one month and monthly injections for an additional month, resulting in a total of 7 injections. With this modified protocol, all eyes achieved complete clinical remission, with a median of 6 injections needed for disease control and no severe complications were reported [16]. Median progression-free and overall survival were 20.8 and 29.3 months, respectively [16]. These results suggest that a shortened MTX regimen can provide comparable local tumor control with fewer injections and potentially fewer treatment-related complications. However, further studies are needed to confirm its long-term efficacy and safety before it can be adopted as a standard treatment approach.

Further evidence supporting the efficacy of intravitreal MTX was provided by Young Gun Park et al. [20] in a study investigating cytokine dynamics in patients with PVRL. The authors demonstrated that intravitreal MTX induced a rapid and significant reduction in aqueous humor IL-10 levels, a biomarker closely associated with disease activity. IL-10 concentrations normalized after a mean of 1.17 months and approximately 8.5 injections, reflecting a favorable therapeutic response [20]. Additional evidence supporting the efficacy of intravitreal MTX was provided by Sou et al. [21], who demonstrated that weekly intravitreal MTX injections were associated with a progressive reduction in aqueous humor IL-10 levels and in the IL-10/IL-6 ratio, both recognized biomarkers of PVRL activity. These results support the efficacy of intravitreal MTX and suggest that intraocular cytokine analysis may serve as a useful adjunct for monitoring therapeutic response.

#### 3.1.2. Rituximab

Rituximab (RTX) (1 mg/0.1 mL) is a monoclonal anti-CD20 antibody and represents an attractive local treatment option for PVRL, given that the vast majority of cases are diffuse large B-cell lymphomas [22]. Compared with MTX, intravitreal RTX offers a targeted mechanism of action and is generally associated with a favorable safety profile, avoiding complications such as corneal epitheliopathy that may occur during prolonged methotrexate therapy [23]. In the largest multicenter retrospective series to date, Larkin et al. [24] evaluated 48 eyes from 34 patients with PVRL treated with intravitreal RTX. Complete remission was achieved in 64.6% of eyes after a median of 3 injections, while partial remission occurred in an additional 22.9%. Rishi et al. [25] further demonstrated that intravitreal RTX was associated with progressive regression of retinal and subretinal lymphoma lesions documented by multimodal imaging, highlighting both the efficacy of the treatment and the value of imaging biomarkers for monitoring therapeutic response.

In a multicenter retrospective study, Kakkassery et al. [26] evaluated 20 eyes from 15 patients with PVRL treated with intravitreal RTX administered on a pro re nata basis. The authors reported a significant reduction in vitreous haze (*p* = 0.0002), accompanied by a significant improvement in visual acuity from a mean of 0.57 logMAR before treatment to 0.20 logMAR during follow-up (*p* = 0.0228). No severe treatment-related complications were reported, supporting the safety of intravitreal RTX as a local therapeutic modality. Earlier reports [27,28] similarly demonstrated favorable outcomes with intravitreal RTX, including regression of vitreous infiltrates and retinal lesions, as well as improvement in visual function. However, local recurrences remain relatively common, and repeated injections are frequently required. For this reason, many centers employ RTX in combination with MTX or as part of a multimodal treatment strategy that includes systemic chemotherapy and, in selected cases, ocular radiotherapy [29].

### 3.2. Systemic Chemotherapy

Systemic treatment of PVRL is largely extrapolated from therapeutic strategies used for PCNSL, reflecting the close biological relationship between these entities and the similarities between the blood–brain barrier and the blood-retinal barrier [30]. High-dose methotrexate (HD-MTX)-based chemotherapy remains the cornerstone of systemic therapy, as it is capable of achieving therapeutic concentrations within the CNS and, to some extent, the ocular compartments [2]. Pharmacokinetic investigations have demonstrated that MTX and cytarabine can penetrate the eye following intravenous administration, with measurable concentrations detected in the aqueous humor and, to a lesser degree, in the vitreous cavity [31,32,33]. Similarly, ifosfamide and trofosfamide have been identified in the aqueous humor after systemic administration, although data regarding their vitreous penetration remain limited [34]. The incomplete and variable penetration of systemic agents into the vitreous has raised concerns regarding the ability of systemic chemotherapy alone to achieve durable local disease control [2].

The role of systemic chemotherapy in the management of PVRL remains controversial, particularly in patients presenting with disease confined to the eye [35]. Although local therapeutic approaches may be adequate for selected cases of isolated unilateral involvement, systemic treatment is more frequently considered in patients with bilateral disease, recurrent ocular lymphoma, or concurrent CNS involvement [36]. Analogous to the treatment paradigm for PCNSL, systemic management of PVRL typically comprises induction and consolidation phases. High-dose MTX-based chemotherapy constitutes the cornerstone of induction therapy, whereas consolidation strategies may include additional chemotherapy, whole-brain radiotherapy (WBRT), autologous hematopoietic stem cell transplantation, or, more recently, targeted agents such as Bruton’s tyrosine kinase inhibitors [36,37].

Advances in the treatment of PCNSL have led to the increasing adoption of the MATRix regimen, which combines HD-MTX, cytarabine, thiotepa, and RTX, particularly in younger patients with good performance status [38]. Long-term follow-up data from the IELSG32 trial demonstrated superior overall survival outcomes with MATRix compared with less intensive MTX-based regimens [39]. Nevertheless, the extrapolation of these results to isolated PVRL should be approached with caution, as only a limited number of patients with ocular involvement were included in the study. Consequently, less intensive HD-MTX-based regimens, administered with or without cytarabine and RTX, are frequently favored in elderly patients or in those with significant comorbidities and reduced functional status [38].

Despite the well-established benefit of systemic chemotherapy in PCNSL, its role in preventing CNS dissemination and improving survival outcomes in patients with isolated PVRL remains insufficiently defined [39]. Several retrospective studies [40,41,42] have reported favorable initial response rates following HD-MTX-based treatment; however, both ocular and CNS relapses continue to occur at substantial rates during long-term follow-up. Moreover, comparative analyses have not consistently demonstrated significant improvements in progression-free survival, overall survival, or the incidence of CNS relapse when systemic chemotherapy is compared with local treatment modalities alone. In some cohorts, systemic treatment has been associated with increased toxicity without a clear survival benefit. Given the rarity of PVRL and the paucity of prospective randomized studies, the precise role of systemic chemotherapy remains incompletely established and continues to represent an important area of ongoing clinical investigation [38].

### 3.3. Radiotherapy

Radiotherapy has long been an important treatment option for PVRL, particularly in patients with localized ocular disease, recurrent lymphoma, or contraindications to systemic chemotherapy [43]. Typical radiation doses range from 30 to 50 Gy, delivered in daily fractions of 1.5–2.0 Gy, with a median dose of 36 Gy associated with the most favorable clinical outcomes [6,11]. Several retrospective studies [40,41,42] have demonstrated high rates of local disease control following ocular or orbital irradiation. In a Japanese multicenter study [43] involving 15 patients with PVRL, radiotherapy was administered at a median dose of 41 Gy, often in combination with chemotherapy. Complete remission was achieved in 13 of the 15 patients, with 2-year overall survival and disease-free survival rates of 74% and 58%, respectively. However, prophylactic cranial irradiation did not appear to reduce the risk of subsequent CNS relapse. Proton beam therapy (PBT) provides highly conformal radiation delivery while limiting exposure of radiosensitive ocular structures. In the study by La Rocca et al. [44], six of 11 patients with localized orbital or ocular adnexal lymphoma received PBT, achieving excellent long-term local control and limited toxicity, with no reported retinopathy or optic nerve injury. However, because patients with PVRL were not included, the applicability of these findings to vitreoretinal lymphoma remains uncertain.

Evidence from retrospective studies suggests that radiotherapy is effective in inducing and maintaining prolonged ocular remission. Berenbom et al. [45] reported no ocular recurrences among patients treated with radiotherapy, either alone or in combination with chemotherapy, whereas relapses occurred in patients managed with chemotherapy alone. Similarly, Milgrom et al. [46] observed favorable outcomes in patients with isolated PVRL treated with HD-MTX-based chemotherapy followed by bilateral orbital irradiation and consolidation with cytarabine, suggesting that combined local and systemic treatment may improve disease control in selected patients. Available studies suggest that ocular radiotherapy provides effective local tumor control with a relatively low incidence of severe vision-threatening complications [44,45,46]. Nevertheless, the current literature is limited by small sample sizes and retrospective study designs, highlighting the need for larger prospective studies to better define the optimal role of radiotherapy in the management of PVRL.

### 3.4. Combined Approaches

Given the high rate of CNS dissemination and the limitations of individual treatment modalities, combined therapeutic approaches have become increasingly common in the management of PVRL [10,12]. Multimodal strategies aim to maximize local disease control within the eye while simultaneously addressing occult CNS involvement and reducing the risk of future neurological relapse [10].

The benefit of combining first-line systemic HD-MTX chemotherapy with local therapy to reduce the risk of CNS relapse remains a matter of debate [10]. This uncertainty stems from the findings of heterogeneous retrospective studies that compared outcomes among patients treated exclusively with local therapy and those receiving systemic treatment alone or in combination with local interventions [9,40,47,48,49,50,51,52,53,54]. In a retrospective study conducted by Hashida et al. [51], where treatment strategies were more uniform, the combination of local therapy and systemic HD-MTX was associated with a significantly prolonged interval before CNS relapse. Kaburaki et al. [53] reported encouraging findings from a small prospective cohort of 11 patients treated according to a regimen similar to that used for PCNSL. The protocol included intravenous RTX, HD-MTX, procarbazine, and vincristine, combined with reduced-dose WBRT (23.4 Gy), high-dose intravenous cytarabine, and intravitreal MTX administration. After a median follow-up period of 40 months, the estimated 4-year cumulative incidence of CNS progression was only 10%. Similarly, the combination of bilateral orbital radiotherapy (30–40 Gy) and systemic HD-MTX yielded promising survival outcomes in a cohort of 11 patients. However, this therapeutic approach was associated with a substantial incidence of radiation-induced ocular toxicity [54].

Although the study had important limitations, including a small sample size, the absence of randomization, and a relatively short follow-up period, Chen et al. [55] demonstrated that systemic chemotherapy-free regimens combined with intravitreal MTX achieved high complete remission rates in patients with isolated PVRL, with the zanbrutinib-RTX combination showing particularly promising progression-free survival and favorable safety profile. In a multicenter study, Soussain et al. [56] showed that high-dose chemotherapy followed by autologous stem-cell transplantation may provide meaningful disease control and prolonged survival in selected patients with relapsed or refractory PCNSL and PVRL. Despite the treatment-related toxicity associated with this intensive approach, the results established autologous stem-cell transplantation as a valuable salvage option for patients with chemosensitive recurrent disease.

Current guidelines [10,12] therefore recommend an individualized treatment strategy based on patient age, performance status, extent of disease, CNS involvement, and anticipated treatment tolerance. In patients who are suitable candidates for intensive therapy, particularly those with bilateral ocular involvement or concomitant CNS disease, a combined systemic and local treatment approach is generally recommended. Conversely, local treatment alone may be a more appropriate option for elderly or frail individuals, patients with substantial comorbidities, or those with isolated ocular lymphoma who are not candidates for systemic chemotherapy [10,12].

## 4. Outcomes and Prognosis

Despite advances in diagnostic techniques and therapeutic strategies, up to 90% of patients eventually develop CNS disease, which represents the principal cause of mortality [11,40]. Most patients initially respond well to local therapies, including intravitreal MTX or RTX, as well as ocular radiotherapy. Complete ocular remission rates exceeding 80–90% have been reported in several studies [33,41,43]. However, local disease control does not necessarily translate into long-term remission, as recurrent ocular disease and CNS progression remain common. Reported median overall survival for patients with isolated PVRL is approximately five years [40,57]. Although retrospective studies have produced conflicting results regarding the superiority of combined approaches, some prospective studies have reported encouraging outcomes. Kaburaki et al. [53] demonstrated that a regimen combining RTX, HD-MTX, intravitreal MTX, and reduced-dose WBRT achieved a 4-year progression-free survival of 74.9% and a 4-year overall survival of 86.3%, with a relatively low relapse rate. Intravitreal therapy offers effective local ocular control but does not prevent CNS disease, while the benefit of systemic HD-MTX in isolated PVRL remains uncertain and must be balanced against toxicity; similarly, autologous transplantation may benefit selected relapsed or refractory patients but lacks comparative evidence and should not be routine first-line therapy. Risk stratification is also limited, as CNS-IPI is not validated for PVRL or secondary vitreoretinal involvement, and molecular markers such as ETV6 and PRDM1 require further validation. Future studies should standardize disease classification and response assessment and prospectively compare local and systemic strategies, incorporating CNS control, visual outcomes, quality of life, and treatment toxicity.

Prognostic factors associated with poorer outcomes include advanced age, bilateral ocular involvement, delayed diagnosis, and the presence of CNS involvement [38]. Recent research has focused on identifying tumor-related biomarkers that may provide prognostic information and assist in monitoring disease activity and treatment response. Among these biomarkers, vitreous IL-10 has emerged as a potential indicator of disease burden. Chan et al. [11] demonstrated a positive association between vitreous IL-10 concentrations and the number of malignant cells present. As an immunomodulatory cytokine produced by malignant B cells, IL-10 may reflect underlying tumor activity. Zhao et al. [58] further observed that elevated vitreous IL-10 levels were associated with poorer clinical outcomes in patients with PVRL. Consequently, serial measurement of IL-10 may serve as a useful prognostic marker, helping clinicians assess disease progression and identify patients who may require more intensive therapy. Future prospective studies are needed to determine whether IL-10-guided monitoring and treatment strategies can reduce or delay CNS progression and improve overall disease management in PVRL [38].

## 5. Emerging Therapies

### 5.1. Targeted Therapy

Bruton’s tyrosine kinase inhibitors (BTKis) have emerged as a potential treatment for PVRL because many patients harbor CD79B and MYD88 mutations, which enhance B-cell receptor signaling and support malignant B-cell survival [59]. Although BTKis are widely used in B-cell lymphomas, their efficacy in PVRL remains under investigation. In a prospective phase II study, Guan et al. [60] evaluated ibrutinib, a first-generation BTKi, as monotherapy in 10 patients with PVRL. After one month of treatment, 90% of patients achieved disease control, including 70% who attained complete remission, and 20% who achieved partial remission, suggesting that BTKis monotherapy is a viable treatment option for PVRL. Gao et al. [61] subsequently compared BTKis monotherapy with combination treatment regimens. While the overall response rates were comparable between the two groups (96% vs. 89%), complete remission was achieved less frequently with monotherapy than with combination therapy (79% vs. 92%). However, severe toxicities occurred more often in the combination therapy group (45%). These findings suggest that combining BTKis with other treatment modalities may improve outcomes, although at the expense of increased toxicity. Despite their therapeutic promise, resistance to BTKis has become an important concern. Studies in activated B-cell DLBCL demonstrated that tumors harboring both MYD88 L265P and CD79A/B mutations responded well to ibrutinib, with an 80% response rate. In contrast, tumors carrying the MYD88 L265P mutation alone, without accompanying CD79A/B mutations, showed resistance to treatment. This observation indicates that MYD88 L265P may contribute to BTKis resistance in certain molecular contexts [62].

Lenalidomide, an immunomodulatory drug with significant antiproliferative activity, has demonstrated efficacy when combined with rituximab in the management of relapsed or refractory PCNSL [63]. In a prospective phase II trial, Ghesquieres et al. [64] evaluated the rituximab-lenalidomide regimen (R2 regimen) in patients with relapsed or refractory PCNSL or PVRL, including 9 patients with PVRL and 10 with secondary PVRL. Complete remission was achieved in 35% of patients, supporting the use of the R2 regimen as a potential treatment alternative for individuals who are not candidates for intensive salvage therapies. Similarly, Zhang et al. [65] investigated a combination of R2 therapy and intravitreal MTX followed by lenalidomide maintenance in 11 patients; however, disease recurrence occurred in 8 cases, including 5 CNS relapses and 3 intraocular relapses. Despite the relatively high relapse rate, the study indicated that the addition of R2 therapy to intravitreal MTX is both safe and clinically effective. Furthermore, monitoring cerebrospinal fluid IL-10 levels may facilitate the early detection of disease recurrence. A recent Chinese multicenter retrospective study found significantly longer progression-free survival with BTKi maintenance than with lenalidomide in patients with PCNSL in remission, including patients previously consolidated with autologous stem cell transplantation [63]. However, imbalances in induction therapy, response status, and ASCT use limit causal interpretation. These findings support prospective evaluation of BTKi as post-remission maintenance, but their ability to prevent CNS or vitreoretinal relapse remains unproven.

The phosphoinositide 3-kinase (PI3K)/protein kinase B (AKT)/mammalian target of rapamycin (mTOR) signaling pathway plays a central role in regulating cellular growth, proliferation, metabolism, and survival, and its aberrant activation has been implicated in the pathogenesis and poor prognosis of PCNSL [66]. Consequently, PI3K and mTOR inhibitors have been investigated as potential therapeutic targets in immune-privileged large B-cell lymphomas [66,67]. Clinical studies evaluating agents such as temsirolimus and buparlisib in relapsed or refractory PCNSL have demonstrated only modest efficacy, with limited progression-free survival (PFS) and overall survival (OS) benefits, as well as a substantial incidence of severe treatment-related adverse events [68]. Nevertheless, preclinical data [69] on novel compounds, including the dual PI3K/histone deacetylase (HDAC) inhibitor BEBT-908, have shown promising antitumor activity, effective blood–brain barrier penetration, and prolonged survival in experimental models. Although current evidence does not support the use of PI3K/mTOR inhibitors as standalone therapies, their incorporation into combination treatment strategies may offer future opportunities to improve outcomes while maintaining an acceptable safety profile [70].

Histone acetyltransferases and histone deacetylases play complementary roles in regulating gene expression through the modulation of histone and non-histone protein acetylation [71]. Disruption of this balance contributes to malignant transformation by affecting cell proliferation, metabolism, and epigenetic regulation. Histone deacetylase inhibitors, such as chidamide, restore acetylation homeostasis, leading to reactivation of tumor suppressor pathways, inhibition of tumor growth, and modulation of the immune microenvironment [72]. Chidamide has shown promising activity in hematologic malignancies and, owing to its ability to penetrate the blood–brain barrier, has emerged as a potential therapeutic option for PCNSL [73]. In a phase II study [74], the combination of chidamide, RTX, and HD-MTX achieved high response rates and favorable tolerability in newly diagnosed PCNSL, supporting the potential role of epigenetic-based combination therapies in immune-privileged large B-cell lymphomas.

Exportin 1 (XPO1) is a key nuclear export protein responsible for transporting proteins and RNA molecules from the nucleus to the cytoplasm [75]. Overexpression of XPO1 has been identified in a variety of malignancies and contributes to tumor progression by disrupting the intracellular localization of tumor suppressor proteins and other regulatory molecules. This has led to the development of targeted therapies aimed at inhibiting XPO1-mediated nuclear export. Selinexor, a selective inhibitor of XPO1, blocks the export of tumor suppressor proteins from the nucleus, promoting cell cycle arrest and apoptosis in malignant cells [76]. Importantly, selinexor is capable of crossing the blood–brain barrier and has already been approved for the treatment of relapsed or refractory DLBCL [77]. Clinical reports have also demonstrated activity against CNS disease, highlighting its potential as a promising therapeutic option for immune-privileged large B-cell lymphomas, including PCNSL and PVRL [78].

The BCL-2 family of proteins plays a central role in regulating apoptosis, with BCL-2 itself acting as a potent anti-apoptotic factor [79]. Overexpression of BCL-2 is frequently observed in lymphoid malignancies, including approximately 60% of patients with PCNSL, suggesting that this pathway represents an attractive therapeutic target. Inhibition of BCL-2 promotes programmed cell death and may enhance the efficacy of other anticancer treatments [80]. Although clinical data on BCL-2 inhibitors in immune-privileged large B-cell lymphomas remain limited, preclinical studies have demonstrated synergistic antitumor activity when agents such as venetoclax or navitoclax are combined with immunochemotherapy [81]. Recent clinical evidence [82] in patients with newly diagnosed DLBCL and high BCL-2 expression has shown encouraging response rates and survival outcomes with venetoclax-based regimens. These findings support the further investigation of BCL-2-targeted therapies as a potential treatment strategy for PCNSL and PVRL.

Interleukin-1 receptor-associated kinase 4 (IRAK4) is a key component of the TLR/MYD88 signaling pathway and plays a central role in the pathogenesis of several hematologic malignancies, including DLBCL [83]. In tumors harboring MYD88 mutations, persistent IRAK4 activation promotes constitutive NF-κB signaling, thereby enhancing cell survival and proliferation [84]. As a result, IRAK4 has emerged as an attractive therapeutic target, particularly in lymphomas characterized by aberrant MYD88-driven signaling. Emavusertib (CA-4948), a selective IRAK4 inhibitor, has shown promising antitumor activity, especially when combined with the BTK inhibitor ibrutinib, with preclinical studies suggesting synergistic induction of apoptosis [85]. An additional advantage of emavusertib is its ability to penetrate the blood–brain barrier, making it particularly relevant for PCNSL and other immune-privileged lymphomas. In a clinical study [86] involving patients with relapsed and refractory PCNSL, the combination of emavusertib and ibrutinib achieved an overall response rate of 50% while maintaining a favorable safety profile, with no treatment-related deaths and predominantly manageable adverse events. These findings support further investigation of IRAK4 inhibition as a targeted therapeutic strategy in immune-privileged large B-cell lymphomas [87].

### 5.2. Immunotherapy

Immune checkpoint inhibitors (ICIs) enhance antitumor immunity by blocking inhibitory PD-1/PD-L1 signaling, thereby restoring T-cell-mediated recognition and destruction of malignant cells [88]. Since alterations involving PD-L1/PD-L2 are frequently observed in PCNSL and primary testicular lymphoma, ICIs have emerged as a promising therapeutic strategy in these diseases [89]. Early clinical evidence has demonstrated encouraging activity of PD-1 inhibitors such as nivolumab, with durable responses reported in patients with relapsed or refractory PCNSL [90]. Similarly, the addition of sintilimab to HD-MTX-based induction therapy achieved high response rates and favorable survival outcomes in newly diagnosed PCNSL [91]. Although these findings support the potential role of ICIs in the management of immune-privileged lymphomas, their use requires careful monitoring because of the risk of immune-related adverse events, including neurological toxicities, which can occasionally result in severe disability or death [70].

Glofitamab is a novel fully humanized IgG1 bispecific antibody with a unique 2:1 configuration, containing two binding sites for CD20 and one for CD3. This design enhances its ability to selectively target CD20-positive B-cell lymphomas while simultaneously recruiting and activating cytotoxic T cells through CD3 engagement, thereby promoting effective tumor cell elimination [92]. In the pivotal phase II NP30180 trial involving patients with relapsed or refractory DLBCL, glofitamab demonstrated substantial clinical activity, achieving an overall response rate of 52% and a complete response rate of 39% supporting its role as an emerging treatment option for aggressive B-cell malignancies [93]. The potential utility of glofitamab in PCNSL was explored by Godfrey et al. [94] in the first reported series of patients with relapsed or refractory PCNSL. Glofitamab was detectable in the cerebrospinal fluid of all evaluated patients, indicating its ability to reach the CNS compartment. Clinical improvement was observed in most treated patients, while treatment-related toxicity remained manageable, with only mild and transient cytokine release syndrome reported. These preliminary findings suggest that glofitamab may represent a promising and well-tolerated therapeutic approach for CNS-involved B-cell lymphomas, although larger studies are needed to confirm its efficacy and safety [70].

Chimeric antigen receptor T-cell (CAR-T) therapy has revolutionized the treatment of relapsed or refractory DLBCL by genetically engineering a patient’s own T cells to specifically recognize and eliminate malignant B cells [95]. Owing to its remarkable efficacy in systemic lymphoma, CAR-T therapy has also been explored in PCNSL and other immune-privileged large B-cell lymphomas [96]. However, its use in these settings has raised concerns regarding immune effector cell-associated neurotoxicity syndrome, as CAR-T cells can migrate into the CNS and potentially induce neurological adverse events [97]. Despite these concerns, emerging clinical evidence has demonstrated encouraging efficacy and an acceptable safety profile in patients with relapsed or refractory PCNSL [98]. In February 2026, the US FDA removed the previous limitation excluding PCNSL from the prescribing information for axicabtagene ciloleucel (Yescarta), permitting its use in eligible adults with relapsed or refractory large B-cell lymphoma involving the CNS; however, isolated PVRL is not listed as a separate indication, and evidence in this setting remains limited. Prospective and retrospective studies have reported overall response rates ranging from approximately 58% to 80%, with a substantial proportion of patients achieving complete remission [99]. Although cytokine release syndrome and neurotoxicity remain important adverse events, most cases are manageable and occur at frequencies comparable to those observed in systemic B-cell lymphoma. Collectively, these findings support CAR-T therapy as a promising therapeutic option for immune-privileged large B-cell lymphomas, while highlighting the need for further research to improve the durability of responses and refine strategies for toxicity prevention and management [100].

## 6. Challenges and Controversies

Given the close relationship between PVRL and PCNSL, all patients with newly diagnosed PCNSL should undergo baseline slit-lamp and dilated fundus examination, followed by periodic ophthalmological surveillance [98]. Neither ocular radiotherapy nor WBRT is recommended as routine prophylaxis: ocular radiotherapy is reserved for documented intraocular disease, whereas WBRT is used as consolidation or salvage therapy in selected patients with cerebral involvement [15]. Although anterior segment optical coherence tomography may prolong disease control in fit patients, ocular relapses may still occur, and its ability to prevent vitreoretinal recurrence remains unproven [10].

Despite significant advances in the diagnosis and management of PVRL, several challenges and controversies remain unresolved. One of the major difficulties is the rarity of the disease, which limits the availability of large prospective studies and randomized clinical trials. Consequently, most treatment recommendations are based on retrospective analyses, small case series, or extrapolation from PCNSL treatment protocols [9,11,12].

The optimal therapeutic approach for isolated PVRL remains a matter of debate (Table 2). While local treatment such as intravitreal MTX, intravitreal RTX, and ocular radiotherapy achieve excellent ocular disease control, they may not prevent subsequent CNS involvement [5,21]. Conversely, systemic HD-MTX-based chemotherapy has been proposed to reduce the risk of CNS dissemination; however, available studies have produced conflicting results regarding its effectiveness in preventing CNS relapse [33]. As a result, there is currently no consensus on whether all patients with isolated PVRL should receive systemic therapy in addition to local treatment. The integration of novel therapies, including BTKis, immunomodulatory agents, immune checkpoint inhibitors, bispecific antibodies, and CAR-T cell therapy, has generated considerable interest [70]. While early clinical results are encouraging, their precise role in PVRL management remains undefined due to limited patient numbers, short follow-up periods, and the absence of comparative studies. Future multicenter prospective trials are essential to establish standardized treatment algorithms, identify patients most likely to benefit from systemic therapy, and optimize long-term disease control while minimizing treatment-related toxicity.

## 7. Conclusions

Despite advances in diagnostic techniques, the management of PVRL remains challenging due to its heterogeneous presentation, high relapse rates, and frequent progression to CNS involvement. These characteristics continue to complicate early diagnosis, risk stratification, and therapeutic decision-making, particularly in the absence of standardized treatment algorithms supported by large prospective trials. Future research should focus on multicenter prospective studies, biomarker-driven risk stratification, and the integration of novel targeted and immune-based therapies to improve long-term outcomes and quality of life for patients with this rare lymphoma.

## Figures and Tables

**Table 1:** Intravitreal methotrexate treatment regimens.

Protocol	Induction	Consolidation	Maintenance
First published protocol [17]	Biweekly until the vitreous is clear of cells (±1 month)	Weekly for 4 weeks	Once a month for 12 months
“Standard” protocol [15]	Biweekly for 4 weeks	Weekly for 8 weeks	Once a month for 9 months
“Clinical response driven” protocol [18]	Biweekly until complete clinical remission *	-	Once a month for 9 months
“Interleukin response driven” LOC network protocol ** [19]	Biweekly for 4 weeks	-	If clinical amelioration and IL-10 < 50% baseline ***: once a month until IL-10 negativation †
Modified protocol [16]	Weekly for one month	Biweekly for one month	Monthly for an additional month

* Clinical remission was defined by the absence of detectable ocular lesions on slit-lamp biomicroscopy, fundus examination, B-scan ultrasonography, and optical coherence tomography [19]. ** This treatment strategy was designed to minimize the cumulative number of intravitreal MTX injections by tailoring therapy according to aqueous humor IL-10 concentrations. Consequently, IL-10 levels are assessed at each treatment visit and during follow-up when disease recurrence is suspected [19]. *** In patients who fail to demonstrate an adequate response during the induction phase, intravitreal MTX is discontinued and alternative salvage therapies are considered within a multidisciplinary setting [19]. † IL-10 < 10 pg/mL. In cases of relapse, the induction phase may be reinitiated [19].

**Table 2:** Comparative efficacy, relapse patterns, and toxicity of current and emerging therapies for PVRL.

Therapy	Current Status	Key Study	Study Design	*N* Patients	Complete Remission (CR)	Median Time to Relapse/PFS	CNS Progression	Major Toxicities
Intravitreal MTX	Standard of care	Frenkel et al. [15] Gao et al. [29]	Retrospective Meta-analysis and systematic review	26	85% [29]	83 % (1-year) 58 % (2-year) [29]	Reported in long-term follow-up	Corneal epitheliopathy, keratopathy, transient IOP elevation, immune-mediated macular edema [101]
Intravitreal RTX	Common adjunct	Larkin et al. [24]	Retrospective	34	>55–65%	Relapses more frequent than MTX monotherapy	No proven CNS protection	Anterior uveitis, vitritis, transient inflammation, occlusive vasculitis [102]
Intravitreal MTX + RTX	Alternative	Multiple institutional cohorts [103]	Retrospective	Variable	Not specifically reported	Superior local disease control than RTX alone	CNS progression remains possible	Kerathopathy, mild intraocular inflammation
Ocular radiotherapy	Alternative	Grimm et al. [40]	Retrospective	Variable	>90%	Often >24 months	35–60%	Cataract, radiation retinopathy *, optic neuropathy, dry eye [104]
HD-MTX	Common systemic option	Multiple retrospective studies [11,12,51]	Retrospective	Variable	70–90%	18–40 months	Lower CNS relapse rates than local therapy alone	Nephrotoxicity, myelosuppression
R-MPV (RTX, MTX, procarbazine, vincristine)	Alternative	Kaburaki et al. [53]	Prospective	11	70–90%	Not reached in small series	10%	Hematologic toxicity, neuropathy
MATRix regimen	Widely used	IELSG studies [39]	Phase II clinical trial	78	70–90%	>2 years	Unavailable	Severe hematologic toxicity
Systemic chemo-free therapy + intravitreal MTX	Alternative	Chen et al. [55]	Prospective	44	93.2%	22.1 months	Yes	Hematological toxicities, cataract, corneal epitheliopathy, elevated IOP
Intensive chemotherapy + autologous hematopoietic stem-cell rescue	Refractory/recurrent PCNSL or PVRL	Soussain et al. [56]	Prospective multicenter trial	43	/	11.6–41.1 months	/	Septic shock, mesenteric necrosis, neurologic toxicity
Ibrutinib	Emerging	Soussain et al. [105]	Clinical trial	44	19%	3.3 months	/	Infection, atrial fibrillation, neutropenia, bleeding, diarrhea
Orelabrutinib + RTX + intravitreal MTX	Emerging	Li et al. [106]	Case report	1	Yes	Not reported	/	Neutropenia, thrombocytopenia, anemia, infection
Lenalidomidă + RTX + intravitreal MTX	Emerging	Zhang et al. [65]	Prospective single-center study	11	90%	12.7 months	/	Neutropenia, thrombocytopenia, anemia, skin rash, venous thromboembolism
CAR-T cell therapy	Experimental	Takahashi et al. [107]	Case report	1	50%	/	/	Cytokine release syndrome, neurotoxicity associated with immune effector cells, prolonged cytopenias, infection

* The risk of radiation retinopathy decreases with lower radiation doses. Nevertheless, Kaushik et al. [104] demonstrated that radiation retinopathy may still develop even at doses below 20 Gy. The risk may increase in patients with pre-existing vascular disorders such as diabetes mellitus [38].

## Data Availability

The original contributions presented in this study are included in the article. Further inquiries can be directed to the corresponding author.

## Abbreviations

PVRL	Primary vitreoretinal lymphoma
DLBCL	Diffuse large B-cell lymphoma
PCNSL	Primary central nervous system lymphoma
CNS	Central nervous system
IL-10	Interleukin-10
EANO	European Association for Neuro-Oncology
MTX	Methotrexate
IL-6	Interleukin-6
RTX	Rituximab
HD-MTX	High-dose methotrexate
WBRT	Whole-brain radiotherapy
IOP	Intraocular pressure
BTKis	Bruton’s tyrosine kinase inhibitors
PI3K	The phosphoinositide 3-kinase
AKT	Protein kinase B
mTOR	Mammalian target of rapamycin
PFS	Progression-free survival
OS	Overall survival
HDAC	Histone deacetylase
XPO1	Exportin 1
IRAK4	Interleukin-1 receptor-associated kinase 4
ICIs	Immune checkpoint inhibitors
CAR-T	Chimeric antigen receptor T-cell
